# Decoding the regulatory roles of circular RNAs in cardiac fibrosis

**DOI:** 10.1016/j.ncrna.2024.11.007

**Published:** 2024-12-09

**Authors:** Qianhui You, Jiajing Yu, Runfang Pan, Jiaming Feng, Haidong Guo, Baonian Liu

**Affiliations:** Department of Anatomy, School of Integrative Medicine, Shanghai University of Traditional Chinese Medicine, Shanghai, 201203, China

**Keywords:** Circular RNAs, Cardiac fibrosis, Biomarker, Therapeutic target, Therapeutic agent

## Abstract

Cardiovascular diseases (CVDs) are the primary cause of death globally. The evolution of nearly all types of CVDs is characterized by a common theme: the emergence of cardiac fibrosis. The precise mechanisms that trigger cardiac fibrosis are still not completely understood. In recent years, a type of non-coding regulatory RNA molecule known as circular RNAs (circRNAs) has been reported. These molecules are produced during back splicing and possess significant biological capabilities, such as regulating microRNA activity, serving as protein scaffolds and recruiters, competing with mRNA, forming circR-loop structures to modulate transcription, and translating polypeptides. Furthermore, circRNAs exhibit a substantial abundance, notable stability, and specificity of tissues, cells, and time, endowing them with the potential as biomarkers, therapeutic targets, and therapeutic agents. CircRNAs have garnered growing interest in the field of CVDs. Recent investigations into the involvement of circRNAs in cardiac fibrosis have yielded encouraging findings. This study aims to provide a concise overview of the existing knowledge about the regulatory roles of circRNAs in cardiac fibrosis.

## 1 Introduction

Globally, cardiovascular diseases (CVDs) stand as the leading cause of mortality. The World Health Organization reports that 31 % of yearly deaths are attributed to CVDs, resulting in 17.5 million fatalities annually [1]. The development of cardiac fibrosis is a common theme in the progression of almost all kinds of CVDs [2]. Cardiac fibroblasts (CFs), a key component of myocardial fibrosis, are in charge of maintaining the balance of the extracellular matrix (ECM) in healthy circumstances [3]. However, when faced with ischemic conditions such as myocardial infarction (MI), characterized by reparative or replacement fibrosis [4] or non-ischemic conditions involving chronic inflammation and edema, which lead to reactive fibrosis [5], the necrosis or apoptosis of cardiomyocytes (CMs) alongside CF activation result in excessive accumulation of ECM [6]. The critical phases are the inflammatory phase, the non-myocyte proliferation phase, and the scar maturation phase [7]. Pathological cardiac remodeling creates distortions in organ structure, reduces cardiac contraction [8], increases the risk of arrhythmogenesis [9], and arises in practically all kinds of cardiovascular disorders, such as hypertrophic cardiomyopathy [10], congestive heart failure [11] and congenital heart diseases [12]. Research on myocardial fibrosis is currently receiving attention. Gibb et al. underlined recent findings on the mitochondrial and metabolism processes involved in myofibroblast differentiation, persistence, and function [13]. Travers et al. emphasized advanced epigenetic regulatory objectives of cardiac fibrosis [14]. However, it remains a challenging issue due to its intricate nature, various clinical situations, considerable tissue heterogeneity, and the constraints of research techniques [15]. In addition to their critical role in neurological disorders [16] and cancer [17], circular RNAs (circRNAs) are implicated in the development of CVDs [18], which are now regarded as promising targets for treating cardiac fibrosis and have become a research hotspot [19].

Initially identified in plant viroids in 1976 [20], circRNAs are single-stranded RNAs generated through pre-mRNA back splicing without the usual 5′ caps and 3’ poly(A) tails of mRNAs. CircRNAs are predominantly situated in the cytoplasm of eukaryotic cells [21], primarily derived from exons, while a few circRNAs originate from introns and are found in the nucleus [22]. With a covalently closed structure that can withstand the degradation of Ribonuclease R, circRNAs are more stable than their homologous mRNA counterparts [23]. Moreover, circRNAs exhibit sequence conservation, and their expression levels are specific to different species [24], tissue [25], and time [26]. With respect to molecular mechanisms of circRNAs in regulating various physiological and pathological processes, circRNAs perform multiple functions: conducting as microRNA (miRNA) sponge [[27], [28], [29]], interacting with protein [[30], [31], [32]], translating peptides [[33], [34], [35]], competing with endogenous mRNAs [[36], [37], [38]], and other newly-discovered mechanisms such as forming circR-loop structure to modulate genome instability [[39], [40], [41]]. The function of circRNAs as miRNAs and protein sponges is well-recognized [42]. They can act as competitive inhibitors to hinder the binding of miRNAs to targeted mRNAs [43]. Furthermore, the combination of circRNAs and RNA binding proteins (RBPs) can regulate gene transcription and translation by suppressing RBP activities [44].

The studies of circRNA in cardiac fibrosis have aroused substantial interest among researchers [45]. The following are recent findings of significant influence on the regulatory roles of circRNAs in cardiac fibrosis. Du et al. suggested and demonstrated that neuroligin gene back-splicing resulted in the production of a novel polypeptide isoform named Nlgn173, which can induce the progression of cardiac fibrosis [46]. Wu et al. discovered that circYAP, downregulating in the heart samples of patients with cardiac hypertrophy, can bind with Tropomyosin-4 (TMP4) and gamma-actin (ACTG) to limit actin polymerization, therefore preventing myocardial fibrosis [47]. Wang et al. proposed that extracellular vesicles (EVs) containing circUbe3a, produced from M2 macrophages, enhanced the G2/M ratio and CF differentiation by specifically impacting the miR-138–5p/RhoC pathway [48]. Yuan et al. discovered that circRNA DICAR prevented pyroptosis in diabetic CMs by binding to valosin-containing protein (VCP) and shielding the mediator complex subunit 12 (Med12) protein from degradation [49]. Wu et al. devised a transgenic cA-circSlc8a1 mouse model, confirming that circSlC8a1 can be transported into mitochondria by interacting with the outer mitochondrial membrane protein to facilitate the production of adenosine-triphosphate enzyme (ATP) [50]. This review summarizes the existing comprehension of the involvement of circRNAs in cardiac fibrosis. In the first part, we discuss the properties, production, and roles of circRNAs. In the second part, we describe the mechanism of cardiac fibrosis, including its characteristics, phases, patterns, influence factors, and therapeutic strategies. In the third part, we compiled the participation of circRNAs in cardiac fibrosis based on their functions in several aspects. Finally, we comment on the problems and possible solutions regarding the fundamental investigation and clinical application of circRNAs in cardiac fibrosis.

## 2 Overview of CircRNA

CircRNAs are single-stranded RNAs with covalently closed ring structures. They are endogenous biomolecules that arise from back-splicing of pre-mRNAs, a process in which a 5′ splice site downstream is combined with a 3’ splice site upstream in reverse order, spanning one or more exons [51]. The lariat model generates an intronic lariat by excluding and pushing out back-spliced exons. Additional back-splicing happens on this lariat, and mRNA is fully assembled when the remainder of the exons arrive. The direct model involves back-splicing to produce a circRNA in the order of priority, which leaves a premature linear RNA with introns [52]. According to various localization within the cell and composition of the sequence, circRNAs can be categorized into four distinct categories: exonic circRNAs (ecircRNAs) [53], intronic circRNAs (ciRNAs) [54], exon-intron circRNAs (EIciRNAs) [55], and mitochondria-encoded circRNAs (mecciRNAs) [[56], [57], [58]]. The majority of endogenous human circRNAs consist of several exons in the cytoplasm, while eIciRNAs and ciRNAs stay inside the nucleus [59]. CircRNAs are generated within the nucleus and are subsequently transmitted to the cytoplasm to perform their specific functions. In the last decade, studies have been conducted on how circRNAs are transported out of the nucleus. These mechanisms include length-related nuclear export [60], nuclear export modulated by N6-methyladenosine (m6A) [61,62], and intercellular trafficking of circRNAs through extracellular exosomes [63,64]. A recent study has discovered a unique process exclusively responsible for transporting circRNA out of the nucleus, necessitating the involvement of Ran-GTP, exportin-2, and insulin-like growth factor 2 mRNA binding protein 1 (IGF2BP1). IGF2BP1 directly binds to circRNAs, recruiting Ran-GTP and exportin-2, demonstrating that the nuclear export of circRNAs is similar to protein export instead of mRNA [65]. Moreover, most circRNAs exhibit long-term stability and extended half-life periods, whose covalently closed ring structures render them impervious to linear RNA decay machinery degradation [66]. Nevertheless, the precise molecular mechanisms underlying the production, cytoplasmic transport, and degradation of circRNAs require further investigation.

**Fig. 1:**
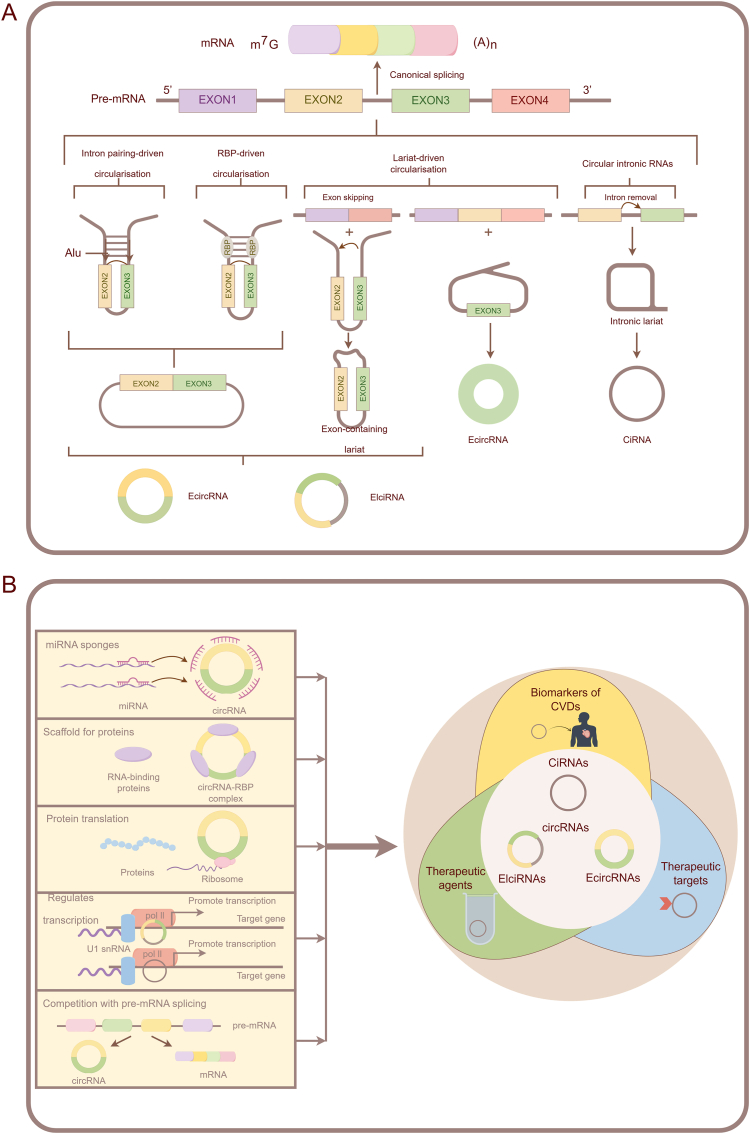
The biogenesis, subclasses, and functions of circRNAs (By Figdraw). (A) The biogenesis and subclasses of circRNAs. Back-splicing produces circular RNAs (circRNAs) with two introns surrounding the exons. Intron pairing-driven circularization employs cis-acting components for pairing surrounded introns. In RBP-driven circularization, this stage is guided by trans-acting elements. In lariat-driven circularization, circRNAs are produced from lariats established during exon-skipping or intron removal events. The above approaches generate EcircRNAs, ElciRNAs, and CiRNAs with distinct sequences. (B) Multiple functions of circRNAs. CircRNAs can function as miRNA sponges, protein scaffolds, and regulators of transcription. They can also compete with endogenous mRNAs and demonstrate translation capability. CircRNAs can act as therapeutic agents, targets, or biomarkers in treating various diseases.

Recent research has revealed tissue-specific, disease-specific, and developmental stages-specific patterns in circRNAs. They are abundant in the brain [67] and human platelets [68], as well as in plants, viruses, and parasites [69]. Mounting evidence suggests circRNAs participate in cancer cell growth, genome instability, drug resistance, and other biological process [70]. From mediating cancer metastasis [71] and regulating neuronal function [72] to modulating the advancement of CVDs such as heart failure (HF) [73] and diabetic cardiomyopathy (DCM) [74], many circRNAs are crucial in the progression of tumors, neurodegenerative and cardiac disorders. CircRNAs’ stability, specificity, and ability to be transported out of cells by exosomes give them enormous potential as biomarkers [[75], [76], [77]]. CircRNAs perform multiple functions: operating as miRNA baits and protein sponges, modulating transcription, and occasionally translating into peptides (Fig. 1). As miRNA-bait, they can compete with endogenous RNAs (ceRNAs) and regulate relative mRNA expression, suppressing free miRNA abundance and preventing the target gene from degradation. In terms of interacting with protein, circRNAs can sequester RBPs [78] and serve as scaffoldings to impact protein-protein interactions [79,80]. Recent data has shown that a class of circRNAs may assist protein localization by acting as recruiters and decoys [81]. For transcription modulation, eIciRNAs, including circEIF3J and circPAIP2, have been linked to the regulation of gene expression transcriptionally or post-transcriptionally through their interaction with the small nuclear ribonucleoprotein U1 (snRNP U1) and RNA polymerase II [82]. Recent research shows that circRNAs can function through novel regulatory processes, including circR-loop formation, m6A modification, guiding RNA editing, and translating polypeptides. CircR-loop has garnered considerable interest due to its participation in orchestrating transcription and exporting circRNA from the nucleus [39]. R-Loop is a unique structure formed during transcription, where DNA and RNA molecules intertwine with non-template single-stranded DNA (ssDNA) [41]. CircRNAs commonly adopt an R-loop structure and coordinate genome instability [83,84]. Additionally, endogenous circRNAs bind to double-stranded RNA-activated protein kinase, inhibiting innate immunity [85,86]. Some circRNAs can also bind with RNA molecules to modulate their function in various pathophysiological processes [[87], [88], [89]]. By combining with mRNA, circRNAs can control their stability and translation [90,91]. Furthermore, certain circRNAs with open reading frames (ORFs) can act as templates for translation, which was first revealed in 2017 [92]. CircRNA translation techniques have emerged as a promising approach in biomedical research [93], such as utilizing circRNAs to translate SARS-CoV-2 receptors, producing the circRNA-RBD-Delta vaccine to tackle the COVID-19 epidemic [94].

In conclusion, the expression of circRNAs is not only abundant and stable but also highly specific, positioning them as promising candidates for ideal biomarkers in disease diagnosis. Furthermore, circRNAs are involved in various facets of disease progression through diverse mechanisms, thereby highlighting their potential as both therapeutic targets and agents.

## 3 The pathogenesis of cardiac fibrosis

Commonly, CFs maintain the balance of ECM, which serves as the framework for CMs, transmits mechanical forces in cardiac tissue, and facilitates electrical conduction [95]. CMs provide forces to control cardiac output and demand continuous energy to sustain their activity and survival. However, in the face of events such as myocardial ischemia and infarction that reduce myocardial tissue perfusion [96], CMs lose an essential source of energy production, and CMs apoptosis or necrosis progresses fibrosis from physiology to pathology [97,98].

Cardiac fibrosis can be identified by two fundamental characteristics: the phenotypic alternation of CFs into myofibroblasts and ECM accumulation, both of which hinder the mechanical-electrical connection of CMs. The increase in passive myocardial stiffness and diastolic dysfunction (DD) is significant in advancing HF [99,100]. Furthermore, inflammation and fibrosis in the perivascular area can reduce the oxygen and nutrients delivered to the tissues while promoting adverse pathological remodeling. Cardiac fibrosis has three phases: the inflammatory phase, the non-myocyte proliferation phase, and the scar maturation phase. The inflammatory phase occurs after an acute event, such as MI, which induces CM necrosis. Immune cells (macrophages, lymphocytes, neutrophils, T cells, and mast cells) infiltrate infarcted tissue and remove dead CMs [[101], [102], [103]]. The increase of various inflammatory mediators activates the transcriptional regulatory axis pattern in the CF population [104,105]. In turn, activated CFs can also modulate the immune response through synthesizing and secreting diverse chemokines, cytokines, and growth factors [106], including Interleukin-6 (IL-6) [107,108], Toll-like receptor 4 (TLR4) [109], and C-C chemokine ligand 5 (CCL5) [110]. By releasing granulocyte-macrophage colony-stimulating factor (GM-CSF), activated CFs can also boost the recruitment of leukocytes [111]. During the non-myofibroblast proliferative stage, granulation tissue is formed, which is populated by activated CFs. This leads to increased ECM production, inflammatory modulators, and adhesion complexes. The acquisition of a myofibroblast phenotype characterizes the scar maturation phase. Myofibroblasts have a more remarkable ability to proliferate, express α-smooth actin (α-SMA), and generate collagen, matrix metalloproteinases (MMPs), periosteal proteins (POSTNs), and tissue inhibitors of metalloproteinases (TIMPs) [112], replacing necrotic CMs with scar formation. The scar is stable and has a high cross-linked collagen content. Collagen I constitutes approximately 80 % of the collagen found in the myocardium, leading to the hardening of the myocardium, and is primarily responsible for cardiac fibrosis. Cross-linking enhances the rigidity of the collagen matrix and impedes its degradation by proteases [113].

**Fig. 2:**
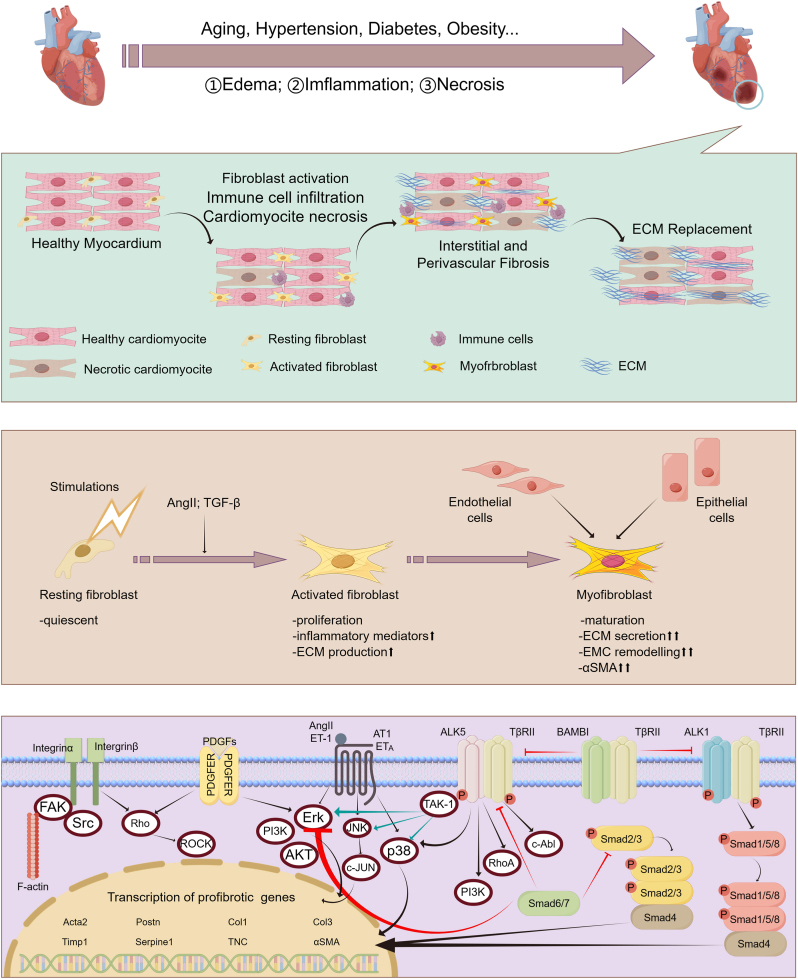
The pathophysiological process of cardiac fibrosis (By Figdraw). Chronic disease risk factors and acute myocardial injuries may result in cardiac fibrosis, triggering edema, inflammation, and necrosis. Cardiac fibroblasts (CFs) are activated to recruit immune cells, secrete inflammatory mediators (inflammation phase), and proliferate rapidly (nonmyocyte-proliferative phase). Myofibroblasts promptly proliferate and express α-SMA and ECM proteins, forming a durable scar (scar maturation phase). A variety of pathways are involved in the regulation of cardiac fibrosis.

Cardiac fibrosis has two basic types: replacement or reparative fibrosis and reactive fibrosis. Both forms of cardiac fibrosis have identical cellular and molecular consequences, but they arise from distinct etiologies and follow separate pathological processes [114]. The etiologies of alternative fibrosis are ischemia conditions, namely acute occurrences, including MI [115,116]. On the other hand, reactive fibrosis is caused by nonischemic situations characterized by persistent inflammation and edema [[117], [118], [119]]. In replacement fibrosis, CMs necrose and are eventually replaced by scar tissue formed by fibroblasts and secreted associated proteins [120]. In reactive fibrosis, there are no necrotic CMs. However, there is still a conversion of fibroblasts to myofibroblasts due to inflammatory signals emanating from a low-grade chronic inflammatory state [74]. ECM proteins accumulate in the intramyocardium and perimyocardium, resulting in interstitial fibrosis [121]. Furthermore, these proteins deposit in the perivascular membrane, leading to perivascular fibrosis. Both forms of fibrosis may manifest concurrently [122]. (Fig. 2).

Clinically, the treatment of HF has commonly involved employing inhibitors of the renin-angiotensin-aldosterone system (RAAS), including angiotensin-converting enzyme inhibitors (Lisinopril, ramipril, enalapril) and angiotensin II receptor antagonists (losartan, candesartan, and valsartan) [[123], [124], [125]]. Nevertheless, existing therapies solely focus on particular pathologic elements and offer restricted overall effectiveness in combating fibrosis. It is necessary to adopt innovative approaches for intervention from multiple viewpoints. An encouraging study discovered that Versican, a CF-originated pro-proliferative proteoglycan, can effectively reduce cardiac fibrosis and promote CM proliferation [126]. The pathways newly revealed to regulate cardiac fibrosis include the Janus kinase/signal transducer and activator of transcription 3 (JAK/STAT3) pathway, c-Jun N-terminal kinase/c-Jun (JNK/c-Jun) pathway [127,128], High-temperature requirement A3-transforming growth factor-beta-insulin-like growth factor binding protein 7 (Htra3-TGF-beta-IGFBP7) axis [129], Protein arginine methyltransferase 5-transforming growth factor-beta pathway (PRMT5-TGFβ) pathway [130], and Phosphoinositide 3-kinase gamma/serum and glucocorticoid-regulated kinase 1 (PI3Kgamma/SGK1) pathway [131]. Moreover, adipokines are considered to be innovative therapies for cardiac fibrosis. A few clinical and preclinical investigations have demonstrated the effectiveness of targeted adipokines, such as pioglitazone, which blocks Ang II-induced myocardial fibrosis through an adiponectin-dependent mechanism and other natural substances and peptides. Nevertheless, obstacles still hinder the application of adipokines as therapeutic medications, such as their high cost and risk of allogenic reactions [132]. Lately, the focus has shifted toward addressing the body's immune system [133]. A recent study discovered that the RNA N6-methyladenosine demethylases (ALKBH5) are responsible for modifying the m6A form of IL-11, which leads to the transformation of macrophages into myofibroblasts and the advancement of myocardial fibrosis [134]. The NLRP3 (Nucleotide-Binding Domain, Leucine-Rich-Containing Family, Pyrin Domain-Containing-3) inflammasome, a complex of various proteins, which is pivotal in activating inflammatory reactions by producing IL-1β and IL-18, also serves as an immune agent that promotes the healing of the cardiovascular system and contributes significantly to tissue scarring [135]. Furthermore, engineering CAR-T cells with lipid nanoparticles that contain mRNA coding for a receptor against the fibroblast activation protein produced by CFs can reduce myocardial fibrosis in mice safely and effectively [136]. The function of peroxisome proliferator-activated receptors (PPAR) in cardiac fibrosis was also elucidated by examining active chemicals, acknowledged stimulants, organic extract chemicals, and nucleic-acid-based medications in various CVD environments [137]. Moreover, ongoing efforts are being made to advance research on non-coding RNAs (ncRNAs) therapies in CVDs. It was established that suppressing the activity of the long noncoding RNA (lncRNA) Gm41724 attenuates myocardial fibrosis by modulating lamina-associated polypeptide 2α (LAP 2α) [138]. The participation of miR-29 downregulation in myocardial fibrosis following MI has been demonstrated. The safety of Remlarsen, a miRNA-29a mimic, was determined throughout the first phase of the clinical investigation [139]. A recent paper suggests that the Microneedle Patch is an appropriate platform for loading microRNA-29 b-containing exosomes to alleviate myocardial fibrosis [140].

In summary, the initiation and progression of cardiac fibrosis represent a multifaceted pathophysiological process. As foundational research advances, the understanding of cardiac fibrosis continues to evolve. Currently, clinical practice predominantly employs pharmacological agents targeting various cardiovascular conditions, such as angiotensin-converting enzyme inhibitors, β-blockers, and Ivabradine for HF; antiplatelet agents (Aspirin and Clopidogrel) and statins for MI. However, a significant deficiency persists in the availability of targeted and effective therapies specifically designed to treat cardiac fibrosis. Nevertheless, with ongoing advancements in both basic and clinical research, the synergistic application of therapeutics addressing heart disease alongside those targeting cardiac fibrosis may yield enhanced therapeutic outcomes for patients.

## 4 The emerging roles of circRNAs in cardiac fibrosis

**Fig. 3:**
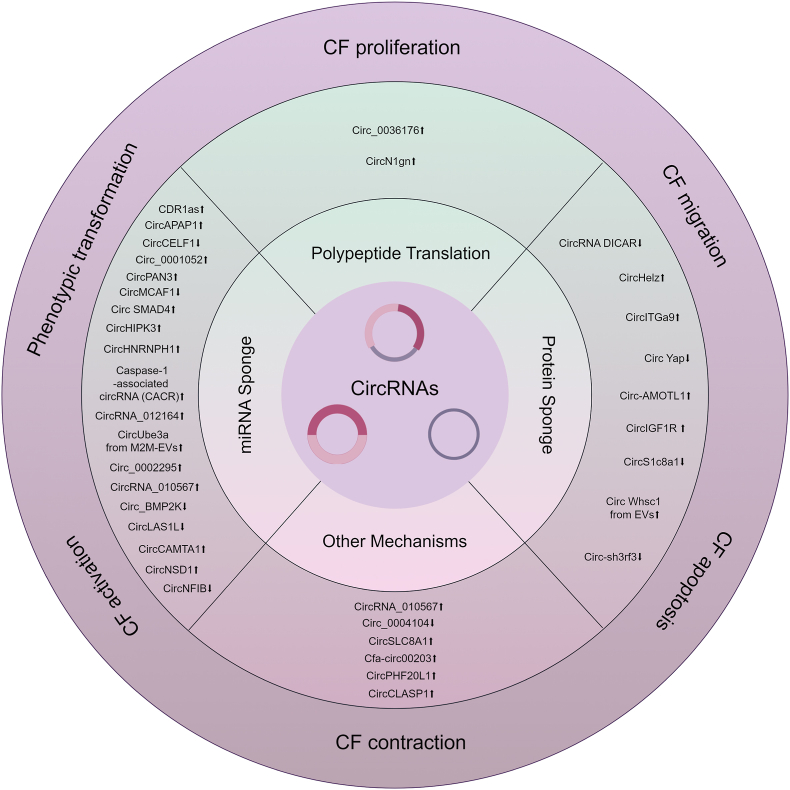
Molecular mechanisms of circRNAs regulating cardiac fibrosis (By Figdraw). CircRNAs modulate cardiac fibrosis through four distinct molecular mechanisms. These upregulated circRNAs exert a pro-fibrotic role, while the downregulated ones protect against cardiac fibrosis. The graph shows multiple circRNAs co-regulate the progression of cardiac fibrosis.

The participation of circRNAs in the pathophysiological mechanisms contributing to cardiac fibrosis is becoming increasingly evident. This section furnishes a summary of the regulatory functions of circRNAs in cardiac fibrosis (Fig. 3, Table 1). It specifically focuses on four molecular mechanisms of circRNAs, including miRNA sponge, interacting with proteins, translating polypeptides, and unknown mechanisms.

**Table 1:** The regulatory roles of circRNAs in cardiac fibrosis.

Research Model	Research Subjects	Intervention Methods (*In vitro*)	Intervention Methods (*In vivo*)	Circular RNA	Effects	Targets or pathways	References
Heart failure	Cardiac fibroblasts, Male C57BL/6 J mice	TGF-β1	Surgical operation (TAC) Isoproterenol	CircSMAD4↑	CF contraction↑, CF activation↑, Media collagen↑, Matrix collagen↑	miR-671–5p↓, FGFR2↓	A. Jeong et al. (2023) [141]
Ischemic heart failure	Pig	None	Bufalin (0.02 mg/kg) Lycorine (0.2 mg/kg)	CDR1as↑	End-diastolic volumes↓, Left ventricular ejection fraction↑, Cardiac fibrosis↓	miRNA-671–5p↓	J. Mester-Tonczar et al. (2020) [142]
Myocardial ischemia-reperfusion injury	Cardiomyocytes, Male C57BL/6 mice	Hypoxia (1 % $O_{2}$)	Surgical operation (LAD ligation)	CircARAP1↑	LVDP ↑, LVSP↓, α-SMA↑, Collagen I↑, Collagen II↑, Apoptosis↑,	miR-379–5p↓, KLF9↑, Wnt/β-catenin pathway↑, Caspase-3↑, Bax↑	X. Li et al. (2023) [143]
Myocardial infarction	Cardiac fibroblasts, Male SD rats	TGF-β1 (10 ng/mL)	Surgical operation (LAD ligation)	CircPAN3↑	CF proliferation↑, Migration↑, Autophagy activity↑	miR-221↓, FoxO3↑, ATG7↑	F. Li et al. (2020) [144]
Acute myocardial infarction	Cardiac fibroblasts, 6-week-old mice	Ang II, M2M-EVs	Surgical operation (LAD ligation), M2M-EVs(1 μg/g)	CircUbe3a from M2M-EVs↑	CF proliferation↑, Migration↑, Phenotypic transformation↑	miR-138–5p↓, Rhoc↓	Y. Wang et al. (2021) [145]
Acute myocardial infarction	Cardiac fibroblasts	TGF-β1 (10 ng/mL), Ang II (100 nM)	None	CircMCAF1↓	CF activation↓, Proliferation↓, Migration↓, α-SMA↓	miR-16–5p↓, SMAD7↓	Y. Wang et al. (2023) [146]
Post-myocardial infarction	Cardiac fibroblasts, NIH/3T3 cells, Male C57BL/6N mice	TGF-β (10 ng/mL)	Surgical operation (LAD ligation)	CircNFIB↓	CF proliferation↓	miR-433↓	Y. Zhu et al. (2019) [147]
Acute myocardial infarction	Cardiac fibroblasts	TGF-β1 (10 ng/mL), Ang II (100 nM)	None	CircLAS1L↓	CF proliferation↓, Migration↓, Apoptosis↑, α-SMA↓, Collagen I↓, Collagen III↓	miR-125 b↓, SFRP5↑	L. ye Sun et al. (2020) [148]
Myocardial infarction	Cardiac fibroblasts, C57BL/6 mice	TGF-β1 (10 ng/mL)	Surgical operation (LAD ligation)	CircNSD1↑	Cardiac function↓, CF proliferation↑, Collagen↑, Fibronectin↑	miR-429–3p↓, SULF1↑, Wnt/β-catenin pathway↑	D. Ji et al. (2024) [149]
Acute myocardial infarction	Cardiac fibroblasts, C57BL/6 mice	Ang II (100 nM or 0–200 nM)	Surgical operation	CircCELF1↓	LVEDD↓, LVESD↓, LVEF↑, LVFS↑, HW/BW↓ CF viability↓, Activation↓, Apoptosis↑, Migration↓	FTO↑, DKK2^m6A^↓, miR-636↓, DKK2↑	X. xun Li et al. (2022) [150]
Diabetic cardiomyopathy	Cardiac Endothelial Cells, C57BL/6 (B6) mice	30 mM glucose (HG)	STZ injection (50 mg/kg)	CircRNA_012,164↑	E/A↓, Cardiac fibrosis↑	miRNA-9-5p↓, Col1a1↑, Fn1↑	H. Wang et al. (2024) [151]
Diabetic cardiomyopathy	Cardiomyocytes, Male C57BL/6 J mice	30 mM glucose (HG)	STZ injection (50 mg/kg)	Caspase-1-associated circRNA (CACR)↑	CM pyroptosis↑, Inflammation↑, Cardiac fibrosis↑	miR-214–3p↓, Caspase-1↑	F. Yang et al. (2019) [152]
Diabetic cardiomyopathy	Cardiac fibroblasts, Male C57BL/6 mice	Ang II (10 μmoL/l)	STZ injection (50 mg/kg)	CircHIPK3↑	Cardiac function↓, CF proliferation↑, Collagen I↑, Collagen III↑	miR-29 b-3p↓	W. Wang et al. (2020) [153]
Cardiac ischemic injury	Cardiac fibroblasts	Hypoxia (1 % $O_{2}$)	None	CircHIPK3↑	CF proliferation↑, Migration↑, Phenotypic transformation↑	miR-152–3p↓, TGF-β2↑	W. Liu et al. (2020) [154]
Postischemic myocardial fibrosis	Cardiac fibroblasts, Cardiomyocytes, Norway rats	TGF-β1 (0,1,5,10 ng/mL)	Surgical operation (LAD ligation)	CircHNRNPH1↑	EF↑, FS↑, LVEDD↑, LVESD↓, LVEDV↓, LVESV↓, α-SMA↓	miR-216–5p↓, SMAD7↑, TGF-β receptor I↓	W. Li et al. W. (2021) [155]
Cardiac fibrosis	Cardiac fibroblasts, C57BL/6 mice	Ang Ⅱ (10 mmol/mL), TGF-β (5 ng/mL)	Ang Ⅱ (1.5 μg/kg/min)	CircHIPK3↑	HW/BW↓, CF proliferation↑, migration↑, Cardiac fibrosis↑, Collagen I↑, Collagen III↑, α-SMA↑	miR-29 b-3p↓	H. Ni et al. (2019) [156]
Cardiac fibrosis	Cardiac fibroblasts, SD rats	Ang II (100 nM)	Ang II (1000 ng/kg/min)	Circ_0002295↑	CF proliferation↓, Migration↓, Cardiac fibrosis↓	miR-1287↓, CXCR2↑	G. Bin Ma et al. (2023) [157]
Cardiac fibrosis	Cardiac fibroblasts	Ang II (0.01 mM)	None	CircRNA_010,567↑	Cardiac fibrosis↑, Collagen I↑, Collagen III↑, α-SMA↑	miR-141↓, TGF-β1↑	B. Zhou et al. (2017) [158]
Cardiac fibrosis	Cardiac fibroblasts	Ang-II (100 nM) TGF-β 1 (10 ng/mL)	None	Circ_BMP2K↓	CF proliferation↓, Migration↓, Apoptosis↑, Collagen I↓, Collagen III ↓, α-SMA↓	miR-455–3p/SUMO1↑	L. Zhang et al. (2020) [159]
Atrial fibrillation	Human atrial fibroblasts, SD male rats	Ang-II	Ang-II (1.5 μg/kg/min)	CircCAMTA1↑	HAF proliferation↑, Collagen I↑, Collagen III↑, α-SMA↑, Acta 2↑, Postn↑, Atrial fibrosis↑	miR-214–3p↓, TGFBR1↑	L. Zhang et al. (2023) [160]
Cardiac hypertrophy	Cardiomyocytes, Male C57BL/6 J mice	Ang II (1 mmol/L)	Surgical operation (TAC)	Circ_0001052↑	Hypertrophic Effects↑, Cardiac fibrosis↑	miR-148a-3p↓, miR-124–3p↓, Srsf1↑ Hipk3↑	M. Yang et al. (2023) [161]
Myocardial infarction	Cardiac fibroblasts, Male C57BL/6 mice	TGF-β (10 ng/mL)	Surgical operation (LAD ligation) Ang II (25 mg/mL)	CircHelz↑	HW/BW↑, EF↓, FS↓, LVIDd↓, LVIDs↓, HYP↑, Cardiac fibrosis↑	NFATc2↑, Nuclear translocation of YAP1↓	P. Pang et al. (2023) [162]
Cardiac hypertrophy	C57BL/6 mice	None	Surgical operation (TAC)	CircITGa9↑	Cardiac function↓, dp/dt (G)↓, LVEF (H)↓, LVFS↓, Collagen↑	Tropomyosin 3↑, Actin polymerization↑	F. Li et al. (2024) [163]
Cardiac hypertrophy	C57BL/6 mice	None	Surgical operation (TAC)	CircYap↓	Cardiac function↑, Cardiac fibrosis↓	TMP4↑, ACTG↑, Actin polymerization↓	N. Wu et al. (2021) [164]
Diabetic cardiomyopathy	T2DM patients, C57BL/KsJ WT, C57BL/KsJ db/db mice	Advanced glycation end-products (200 μg/mL)	None	CircRNA DICAR↓	LVESV↑, LVEDV↑, LVEDV↑, EF↓, FS↓, CMs pyroptosis↓, Cardiac fibrosis↑, Cell hypertrophy↑	DICAR-VCP-Med12 degradation↓, DICAR-JP↓	Q. Yuan et al. (2023) [165]
Diabetic cardiomyopathy	Cardiac fibroblasts, Male C57BL/6 J mice	30 mM glucose (HG)	STZ injection (50 mg/kg)	Circ-AMOTL1↑	FS↑, EF↑, Cardiac fibrosis↓, CF proliferation↓, Collagen I↓, Collagen III↓, α-SMA↓, ROS↑, Reactive nitrogen species↑	TGF-β1↓, EIF4A3↑, MARCKS stability↑	Y. Yang et al. (2023) [166]
Cardiac hepatopathy	C57BL/6 J mice	None	Surgical operation (TAC)	CircSlc8a1↓	Congestive heart failure↑, Peripheral edema↑, Body weight↑, LVEF↓, LVFS↓, dp/dt↓, LVEDD↑, LVESD↑	CircSlc8a1 translocation↑, ATP synthesis↑	N. Wu et al. (2023) [167]
Hypoxia	Cardiomyocytes, Male C57BL/6 J mice, Male SD rats	None	Surgical operation	CircWhsc1 from EVs↑	CM proliferation↑, G2/M ratio↑, Angiogenesis↑, Cardiac fibrosis↓	TRIM 59/STAT 3/Cyclin B2 pathway↑	G. Wei et al. (2022) [168]
Cardiac ischemic injury	Cardiomyocytes, ICR mice	None	Surgical operation	CircIGF1R↑	CM proliferation↑, Apoptosis↓, Cardiac dysfunction↓, Cardiac fibrosis↓	DDX5↑, β-catenin↑, cyclin D1↑, c-Myc↑	T.-K. Shan et al. (2024) [169]
Cardiac fibrosis	Cardiac fibroblasts, ICR mice	TGF-β1 (5 ng/mL), ISO (10 μM)	ISO (30 mg/kg/day)	Circ-sh3rf3↓	Cell size↑, Stress fibers↑, CF phenotypic transformation↓	GATA-4↓, miR-29a↑	C. X. Ma et al. (2023) [170]
Heart failure	Human atrial fibroblasts, Mouse cardiac fibroblasts	Ang II	None	Circ_0036,176↑	CF proliferation↓	Myo 9a-208↑, Cyclin/Rb pathway↓	J. Guo et al. (2022) [171]
Heart failure	C57BL/6 J mice	None	Surgical operation	CircNlgn↑	Cardiac function↓, Collagen↑, CF proliferation↑, CM viability↓	Nlgn173↑, ING 4↑, C8 orf 44-SGK 3 promoter↑	W. W. Du et al. (2021) [172]
Cardiac fibrosis	AC cells, Cardiac fibroblasts, Cardiomyocytes, C57BL/6 J mice	Doxorubicin (0.1 mM, 0.25 mM)	Doxorubicin (3 mg/kg)	CircNlgn↑	LVEF↓, LVFS↓, dp/dt↓, CF proliferation↑, Collagen I↑, Collagen III↑	TGF-β1↑, CTGF↑, Fibronectin↑, Vimentin↑, Gadd45 b↑, Sema4C↑, RAD50↑, p38↑, pJNK↑, Nlgn173↑, H_2AX↑, IL-1b↑, IL-2Rb↑, IL-6↑, EGR1↑, EGR3↑	J. Xu et al. (2022) [173]
Acute myocardial infarction	Male SD rats	None	Surgical operation	CircRNA_010,567↑	Cardiac function↑, LVEF↑, LVFS↑, LVDd↓, LVDs↓, Cardiac fibrosis↓, Apoptosis↓	TGF-β1↓	M. Bai et al. (2020) [174]
Cardiac hypertrophy	SPF C57BL/6 mice	None	Surgical operation (TAC)	mmu-Nfkb1_0001↑, mmu-Hecw2_0009↑, mmu-Itgbl1_0002↑, mmu-Cpeb3_0007↓, mmu-Ryr2_0040↓	EF↑, FS↑, LV mass↑, IVSd↑, LVPW↑, LVIDd↑, LVPWd↑, Collagen↑, Cardiac fibrosis↑	mmu-Hecw2_0009-miR-346–3p/Cthrc1/Fmod/Inhbb/Orai2/Cemip↑, mmu-Cpeb3_0007-miR-92a-2-5p-Cacna2d2/Cdh8/Kcnj3↓, mmu-Ryr2_0040-miR-103-3p-Celsr2/Prmt8↓	Y. Chen et al. (2022) [175]
Persistent atrial fibrillation	6 persistent, 6 paroxysmal AF patients	None	None	Circ_0004104↓	None	TGF-β1↑, MAPK↑	Y. Gao et al. (2021) [176]
Atrial fibrillation	Atrial fibroblasts, Mongrel dogs	Ang Ⅱ (5.0 μmol/L)	Electrical stimulation (5 V, 0.2 ms, 500 bpm)	Cfa-circ 00203↑	Atrial fibroblast proliferation↑, Migration↑, Apoptosis↓, α-SMA↑, Collagen I↑, Collagen III↑	MMP2↑, MMP9↑, Bax↓, Caspase 3↓	W. Shangguan et al. (2023) [177]
Cardiac fibrosis	Cardiac fibroblasts	TGF- β1 (10 ng/mL)	None	mmu_-circ_0001020↑, mmu_-circ_0000512↑, mmu_-circ_0001204↑, mmu_-circ_0001645↓, mmu_-circ_0012,530↓, mmu_-circ_0001632↓	CF proliferation↑, Cardiac fibrosis↑, α-SMA↑, Collagen I↑	miR-34a-5p↑, miR-15↑, let-7d-5p↓, TGF-β pathway, AMPK pathway, PI3K-Akt pathway	X. Gu et al. (2020) [178]
Diabetic cardiomyopathy	C57BL/6 mice, C57BL/6 db/db mice (fasting blood glucose levels ≥11.1 mmol/L)	None	None	CircPHF20L1↑, CircCLASP1↑, CircSLC8A1↑	Average weight↑, Average ratio of heart weight/tibia length↑, LVIDd↑, LVIDs↑, FS↓ EF↓, E/e’ ratio↑, Collagen deposition↑	CircCLASP1/miR-182–5p/Wnt7a↑, CircSLC8A1/miR-29b-1-5p/Col12a1↑, CircPHF20L1/miR-29a-3p/Col6a2↑	L. Yuan et al. (2024) [179]

### 4.1 MicroRNA sponge

Numerous miRNAs have been identified as agents that can either augment or inhibit the activation of CFs across various physiological and pathological states [[180], [181], [182]]. CircRNAs regulate the advancement of cardiac fibrosis by interacting with miRNAs involved in adverse cardiac remodeling. In the pathological model of HF, Anna Jeong et al. reported that the circSMAD4-miR-671-5p-FGFR2 axis participated in the phenotypic transformation of CFs [141]. Mester-Tonczar et al. effectively detected circRNA CDR1as in the pig model and found a strong correlation between CDR1as and improved left ventricular (LV) function. This association was observed while using anti-fibrotic therapy with the natural substances bufalin and lycorine. Vivo experiments confirmed that circRNA CDR1as exerted anti-fibrotic effects by sponging miRNA-671–5p [142]. In the pathological environment of MI, Li et al. assessed the pro-fibrotic role of circARAP1 in myocardial ischemia-reperfusion injury (MI/RI). CircARAP1 expression was notably elevated in hypoxia/reoxygenation-triggered CMs. Gain-of-function approaches uncovered that circARAP1 exacerbated cardiac fibrosis by modulating the miR-379–5p/KLF9 pathway to promote the Wnt/β-catenin pathway [143]. Additionally, Li et al. discovered that circPAN3 contributed to autophagy-mediated myocardial fibrosis through miR-221/FoxO3/ATG7 pathway [144]. Wang et al. suggested that M2 macrophage-originated circUbe3a in small extracellular vesicles facilitated CF growth, migration, and differentiation by sponging miR-138–5p to repress RhoC transcriptionally [145]. Moreover, Wang et al. clarified the anti-fibrotic role of circMACF1 in the activation of CFs following MI. Mechanism studies showed that circMACF1 alleviated CF activation, migration, and growth through miR-16–5p/SMAD7 axis in TGFβ1-induced fibrosis [146]. Zhu et al. found that circNFIB was significantly reduced in the MI murine model, which protected against myocardial fibrosis by sponging miR-433 to attenuate CF proliferation [147]. Additionally, Sun et al. discovered Circ_LAS1L, decreasing in the heart of MI patients, hindered CF proliferation and migration by sponging miR-125 b to promote SFRP5 (Secreted frizzled-related protein 5) [148]. Ji et al. investigated the pro-fibrotic role of circNSD1, which enhanced the expression of Sulfatase 1 (SULF1) and the activation of the Wnt/β-catenin axis by sponging miR-429–3p [149]. Li et al. elucidated the anti-fibrotic role of circCELF1 in MI mice. Mechanism research showed that CircCELF1 upregulated the expression of Dickkopf-2 (DKK2) by sponging miR-636. DKK2-m6A level was also reduced by circCELF1 via transcriptionally suppressing FTO [150]. In the DCM model, Wang et al. validated the increased expression level of circRNA_012,164 and decreased level of miRNA-9-5p in both in vivo and in vitro experiments. CircRNA_012164/miRNA-9-5p axis modulated the transcriptional activity of genes implicated in CF proliferation, and its silencing exhibited protective effects on cardiac endothelial cells [151]. Yang et al. found that caspase-1-associated circRNA (CACR) was remarkably elevated in the serum of the DCM patients. CACR sequestered miR-214–3p to activate caspase-1, triggering CM pyroptosis [152]. Wang et al. observed that circHIPK3 knockdown attenuated myocardial fibrosis in the DCM murine model. Functionally, circHIPK3 played a pro-fibrotic role during DCM by interacting with miR-29 b-3p [153]. In the model of cardiac ischemic injury, Liu et al. demonstrated that CircHIPK3, remarkably increasing in CFs in hypoxia condition, suppressed the expression level of miR-152–3p to upregulate TGF-β2 expression and ameliorated CF growth, migration, and differentiation [154]. Li et al. found that circHNRNPH1 was markedly elevated in the myocardium sample after MI. The mechanism study showed that circHNRNPH2 hindered myocardial fibrosis by sponging miR-216–5p to promote the expression level of SMAD7, accelerating the degradation of TGFβ receptor I (TGFBR1) [155]. In the model of cardiac fibrosis, Ni et al. observed that circHIPK3 expression was remarkably upregulated in Ang II-cultured CFs. Mechanism studies showed that inhibition of circHIPK3 alleviated the production of collagen I, collagen III, and α-SMA and prevented myocardial fibrosis by interacting with miR-29 b-3p [156]. Ma et al. discovered that circ_0002295 and CXCR2 were overexpressed, and miR-1287 was downregulated in heart samples of patients with cardiac fibrosis. Circ_0002295 facilitated the growth and migration of Ang II-treated CFs by sponging miR-1287 to promote CXCR2 expression [157]. Moreover, a study by Zhou et al. revealed that circRNA_010,567 was remarkably elevated in the murine model. As bioinformatics analysis predicted, circRNA_010,567 combined with miR-141, a direct target of TGF-β1 [158]. Zhang et al. uncovered the anti-fibrotic effects of circ_BMP2K, which promoted the modulation of miR-455–3p on its downstream gene SUMO1 to block CF proliferation and migration [159]. In the pathological environment of atrial fibrillation (AF), Zhang et al. observed that circCAMTA1 expression ascended in samples of atrial muscle taken from AF patients. Functionally, circCAMTA1 aggravated Ang-II-induced cardiac fibrosis both in vitro and in vivo by counteracting the inhibitory impact of miR-214–3p on TGFBR1 expression [160]. In the model of cardiac hypertrophy, Yang et al. found that circ_0001052 played a pro-fibrosis role in Ang II-treated CMs. Mechanism studies showed that circ_0001052 promoted Hipk3 expression by binding with miR-148a-3p and miR-124–3p while additionally recruiting Srsf1 [161].

Investigations into the mechanisms elucidating the process by which circRNAs sponge miRNA indicate that these molecules possess significant potential as therapeutic targets or biomarkers in cardiac fibrosis. Moreover, as the database of predicted circRNAs’ interactions with potential miRNAs continues to expand, and as both in vivo and in vitro technologies for modulating the expression of specific circRNAs and miRNAs advance, the biological function of circRNAs through sponging miRNAs to regulate targeted genes remains the most extensively investigated molecular mechanism.

### 4.2 Interacting with proteins

Besides engaging with RNA molecules, circRNAs can also sequester or combine with proteins to regulate their function or localization in the progression of myocardial fibrosis. In the pathological model of MI, Pang et al. reported the elevation of circHelz in heart samples of MI murine models and TGFβ-treated CFs. Through the process of attaching to YAP and promoting its transportation to the nucleus, circHelz was upregulated in response to the nuclear factor of activated T cells, cytoplasmic 2 (NFATc2), acting as a transcriptional activator to exacerbate CF proliferation [162]. In the pathological model of cardiac hypertrophy, according to research by Li et al., circITGa9, a circRNA that was overexpressed in cardiac tissues of patients, developed myocardial fibrosis through interacting with tropomyosin 3 (TPM3) and controlling actin polymerization [163]. Moreover, Wu et al. showed that in pressure overload mice and the hearts of patients, circRNA YAP was markedly downregulated. It was discovered that TMP4 and ACTG engaged with circYap to enhance TPM4's association with ACTG, which attenuated actin polymerization and fibrosis [164]. In the pathological setting of DCM, Yuan et al. demonstrated that circRNA DICAR was notably increased in the heart samples of diabetic murine models. They verified that DICAR overexpression prevented CM pyroptosis in DICAR gene-modified mice and vivo-cultured and vitro-cultured CMs. The fundamental molecular process may involve DISCAR binding to VCP to prevent MED12 from degrading [165]. Furthermore, Yang et al. discovered that in an in vivo murine model of diabetes produced by streptozotocin (STZ), there was a substantial upregulation of circ-AMOTL1 expression. Vitro tests revealed that circ-AMOTL1 silencing hampered CF proliferation and decreased ROS and NO levels in high glucose environments. By interacting with EIF4A3 (Eukaryotic initiation factor 4 A-III) to increase the MARCKS stability, circAMOTL1 promoted cardiac fibrosis [166]. In the pathological environment of cardiac hepatopathy, Wu et al. constructed genome-modulated mice for antisense-mediated circRNA (cA-circSlc8a1) knockdown without affecting the parental linear RNA, which triggered congestive heart failure and cardiac hepatopathy. In terms of mechanism, they identified that circSlc8a1 was transported into mitochondria to promote ATP production [167]. In hypoxia, Wei et al. revealed that heart regeneration could be induced by circWhsc1, which was substantially increased in cardiac endothelial cells (ECs) and EC-derived EVs. Mechanism research demonstrated EV-derived circWhsc1 phosphorylated Tripartite Motif Containing 59 (TRIM59) in greater quantity, strengthening TRIM59's combination with STAT3, phosphorylated STAT3, and accelerated CM proliferation [168]. In the model of cardiac ischemic injury, Shan et al. observed that the expression of circIGF1R was elevated in both in vivo and in vitro experiments. RNA pull-down Western blot and RNA immunoprecipitation showed that the activation of the circIGF1R/DDX5/β-catenin axis further upregulated the transcriptional level of cyclin D1 and c-Myc, promoting the regeneration and repair of heart tissue following ischemic damage [169]. In the model of myocardial fibrosis, Ma et al. found that circ-sh3rf3 expression declined in rats given isoproterenol during the phenotypic transformation of CFs. They more thoroughly verified that circ-sh3rf3 may decrease fibroblast-myofibroblast development and cardiac fibrosis by reducing GATA-4 expression and upregulating miR-29a expression [170].

These studies indicate that circRNAs can modulate specific proteins’ function or subcellular localization by interacting with polypeptides, thereby playing a pivotal role in the pathophysiological mechanisms underlying various diseases. A variety of circRNAs act as protein scaffolds, exerting a significant influence on the progression of cardiac fibrosis by either enhancing or inhibiting the function of one or more proteins. Furthermore, with the development of circRNA-specific probes and other technologies related to circRNA-proteins interaction, the broader biological functions of circRNAs through interacting with proteins will be discovered, providing support for clarifying the functions and clinical applications of circRNAs.

### 4.3 Polypeptide translation

Researchers have investigated the possibility of circRNAs acting as translation templates in addition to their non-coding roles [183]. The linear mRNA translation requires the structures of 5′ caps and 3′ poly(A) tails [184]. CircRNAs can only be translated cap-independently via the internal ribosome entry site (IRES) [[185], [186], [187]] or by m6A [[188], [189], [190]], owing to their covalent ring structure. Nevertheless, the specific characteristics of circRNA translation and the precise processes regulating the translation of circRNAs are still largely unexplored [191]. Ribosome profiling, or RIBO-seq, employs high-throughput sequencing of mRNA fragments protected by ribosomes. This technique monitors translation with exceptional speed, accuracy, and scalability [192]. As RIBO-seq advances, translated circRNAs are increasingly discovered in various organisms. A substantial number of translatable circRNAs, over 200 in total, have been reported in recent investigations [193,194]. Current research on circRNA translation of polypeptide in cardiac fibrosis is presented as follows. In the model of HF, according to research by Du et al., the myocardium of HF patients had higher expression levels of circ_0036,176, a circRNA derived from the Myosin IXA (Myo9a) gene. Additionally, it was found to be abundant in exosomes of CMs that showed overexpression of circ_0036,176, which resulted in the downregulation of CF proliferation. Surprisingly, the genome circ_0036,176 harbored an IRES element and an ORF of 627 nucleotides that coded for Myo9a-1608. MiR-218–5p inhibited Myo9a-208 expression by interacting with circ_0036,176, activating the cyclin/Rb pathway. By encoding the Myo9a-208 and inhibiting the cyclin/Rb pathway, circ_0036,176 prevented the proliferation and growth of CFs [171]. Moreover, Du et al.‘s study demonstrated that circular neuroligin RNA (circNlgn) contributed to cardiac remodeling. Chromatin immunoprecipitation sequencing was conducted on cardiac tissues from patients with congenital heart diseases, such as tetralogy of Fallot and mitral stenosis, which showed a considerable increase in circNlgn expression. A circNlgn-transgenic murine model was developed and showed deteriorated cardiac function. Echocardiography demonstrated downregulated left ventricular fractional shortening (LVFS), left ventricular pressure (dp/dt), and left ventricular ejection fraction (LVEF). Reduced fibrosis markers and hindered cardiac fibrosis levels were observed when endogenous circNlgn was silenced. Functionally, the neuroligin gene was back-spliced to produce Nlgn173 protein with a 9-amino-acid motif to localize in the nucleus. Nlgn173 reached the nucleus due to LaminB1 attaching its nuclear localization motif. According to the chromatin immunoprecipitation study, Nlgn173 was connected to the promoters of inhibitor of growth protein 4 (ING4) and serum and glucocorticoid-inducible kinase-3 (C8orf44-SGK3). This interaction resulted from excessive collagen deposition, elevated CF proliferation, and reduced CM viability. The abnormally controlled expression of ING4 and C8orf44-SGK3, along with the nuclear translocation of Nlgn173, was confirmed by a group of 145 patient samples [172]. The function of circNlgn is further investigated in doxorubicin-induced cardiac fibrosis. Doxorubicin is a widely employed chemotherapeutic agent for the treatment of multiple cancer types, albeit it is accompanied by adverse effects such as emesis, alopecia, and myelosuppression. Cardiomyopathy, cardiac fibrosis, and HF are the primary adverse effects since doxorubicin inhibits DNA replication and produces cytotoxicity. Xu et al. overexpressed circNlgn in transgenic mice and found circNlgn induced cardiac fibrosis by activating Growth arrest and DNA-damage-inducible beta (Gadd45 b), Semaphorin 4C (Sema4C), RAD50, p38, and pJNK. Functional studies showed that circNlgn interacted with H2AX to generate gH2AX, leading to the upregulation of IL-1b, IL-2Rb, IL-6, Early Growth Response 1 (EGR1), and Early Growth Response 3 (EGR3), eventually triggering CM apoptosis, increased CF proliferation, and collagen disposition. The study indicated the potential of circNlgn as a therapeutic target to mitigate cancer treatment-induced cardiotoxicity [173].

The role of circRNA in polypeptide encoding significantly advances our comprehension of protein translation and unveils novel avenues for the exploration of circRNA and non-coding RNA. Nonetheless, research into circRNAs’ function of translating polypeptides remains limited. This limitation may stem from the technical challenges associated with screening for circRNAs with translation capabilities, identifying translation products, and quantifying these outputs. However, with the rapid advancements in artificial synthesis technologies for circRNAs in vitro environment, a deeper understanding of the biological roles played by circRNA-derived polypeptides in cardiac fibrosis and other diseases will be further revealed. Moreover, the function of circRNAs translating into polypeptides offers significant support for the development of circRNA vaccines and artificial protein engineering.

### 4.4 Mechanism unknown

Numerous circRNAs’ functions in cardiac fibrosis are studied only for macroscopic effects or associated influence pathways. The specific molecular mechanisms that are responsible for these functions have not been entirely comprehended. In the model of MI, a study conducted by Bai et al. revealed that circRNA 010567 siRNA enhanced cardiac function, alleviated myocardial fibrosis, and inhibited CM apoptosis, thereby further blocking MI-induced myocardial fibrosis. The underlying mechanism was attributed to inhibiting the TGF-β1 pathway [174]. In the pathological environment of CH, Chen et al. reported possible circRNAs, such as mmu-Nfkb1_0001, mmu-Hecw2_0009, and mmu-Itgbl1_0002, may be promising targets for treating CH-induced fibrosis based on gene set enrichment analysis (GSEA) [175]. In the model of AF, Gao et al. found that hsa_circ_0004104 exerted a pro-fibrotic role by regulating MAPK and TGF-β pathways and, therefore, could be a promising biomarker in AF persistency [176]. Furthermore, Shangguan et al. discovered that in both canine models and in vivo settings of AF, cfa-circ002203 was elevated. Cfa-circ002203 overexpression weakened CF apoptosis while promoting migration and proliferation. Western blotting demonstrated decreased expression of Bax and Caspase 3 and increased levels of α-SMA, collagen I, collagen III, MMP2, and MMP9 [177]. In the model of cardiac fibrosis, Gu et al. developed a vitro TGF-β1-treated mouse model and examined the expression profile of circRNAs. The entirety of 283 circRNAs were detected as abnormally regulated in CFs, with 79 increased and 204 decreased. A network of ceRNA was constructed, revealing that these mRNAs were highly concentrated in signaling pathways such as the TGF-β, MAPK, AMPK, and PI3K-Akt. The identified ceRNAs and bioinformatics evaluations have indicated the probable involvement of circRNAs in cardiac fibrosis, offering valuable insights into comprehending the underlying mechanism and aiding in identifying therapeutic agents or targets of cardiac fibrosis [178]. In the model of DCM, Yuan et al. constructed the profile of circRNA expression in cardiac fibrosis in the type 2 DCM model by utilizing echocardiography and techniques of real-time quantitative polymerase chain reaction (RT-qPCR) and DNA microarray to measure db/db mice. A circRNA-miRNA-mRNA network was constructed and illustrated that CircPHF20L1, circCLASP1, and circSLC8A1 might be critical in the development of DCM-fibrosis and CircPHF20L1/miR-29a-3p/Col6a2 might be a significant pathway [179].

These studies suggest that circRNAs may influence cardiac fibrosis through alternative molecular mechanisms. However, comprehensive investigations are essential to elucidate the specific molecular mechanisms by which these circRNAs contribute to cardiac fibrosis. Furthermore, there is a notable absence of studies examining the role of circRNAs in modulating cardiac fibrosis through interactions with chromosomal DNA or competition with homologous mRNA, warranting further exploration in future research.

## 5 Conclusions and perspectives

A substantial number of the studies mentioned above serve as evidence of the significant involvement of circRNAs in the pathophysiological mechanisms underpinning myocardial fibrosis. The diverse range of functions fulfilled by circRNAs indicates their potential as biomarkers, diagnostic targets, or therapeutic agents for diagnosing cardiac fibrosis.

From the perspective of clinical application, there is only one clinical study on circRNAs in cardiovascular diseases on Clinicaltrials.gov and PubMed Clinical Trial. The study demonstrated the diagnostic potential of circRNA-Uck2 in adults with acute myocardial infarction (AMI) using microarray analysis and real-time polymerase chain reaction (PCR) in AMI animal models, and small samples of AMI patients and the ability of circRNA-Uck2 to distinguish adults with AMI from patients with unstable angina pectoris was also evaluated (NCT03170830). However, regrettably, no clinical studies on the regulatory roles of circRNAs in cardiac fibrosis were identified. The clinical application of circRNAs in research on CVDs and cardiac fibrosis is currently limited, highlighting the need for further investigation in both fundamental and clinical research. The obstacles and possible solutions in the investigation of circRNAs and research on cardiac fibrosis are outlined below.

Firstly, detecting individual circRNAs is technically challenging due to the sequence overlap between them and corresponding mRNAs. Additionally, circRNAs are minimally expressed under normal conditions, which can restrict their therapeutic utility as a biomarker. To tackle this problem, the rapid advancement of sequencing technology in recent years offers significant benefits in detecting circRNA. With the declining price of sequencing, there will be more emphasis on the potential and value of circRNAs as dependable diagnostic biomarkers and therapeutic targets of novel therapeutic strategies. A promising area of research is creating methods for profiling circRNAs at the single-cell level, as numerous circRNAs are explicitly expressed depending on the cell type. Several studies have identified circRNAs in individual cells by utilizing random primers linked to fixed anchor sequences or by integrating microfluidics with strand-specific total RNA sequencing. However, these techniques are effective only for circRNAs in high abundance and require further refinement to be applicable for broader circRNA analysis. Moreover, Simultaneous single-molecule imaging within living cells or tissues facilitates the visualization of individual RNA molecules in a sample.

Secondly, it is technically challenging to knock down or overexpress a particular circRNA. The siRNA/shRNA designed for the BSJ overlaps the linear mRNA, potentially resulting in substantial off-target effects. The CRISPR/Cas13 system demonstrates a higher specificity in the knockout of the target gene compared to siRNA. One promising approach involves utilizing catalytically inactive forms of Cas13 to target the BSJ. By employing fluorescently labeled gRNAs, researchers can monitor circRNAs within live cells. Attaching RBPs or RNA-modifying enzymes can also obstruct binding sites or introduce new functionalities. The precise attachment of RNA-modifying enzymes will serve as a valuable method for further understanding how modifications like m6A influence the stability and functionality of circRNAs. Nevertheless, the efficacy of this approach in the human body necessitates additional research. In addition, the overexpression of circRNAsis is also demanding, especially for larger circRNAs. The overexpression of circRNAs is heavily dependent on plasmid expression. Therefore, Sanger sequencing is recommended for BSJs of expressed products to ensure the accuracy of overexpressed circRNAs, especially for certain circRNAs that rely on their specific BSJs.

Some recent advances in circRNAs and related technologies are gradually solving the problems in previous research. Advancements in RNA synthesis and RNA vaccination technologies have served as valuable resources for the in vitro synthesis of circRNAs and circRNA-based therapies. Furthermore, an efficient delivery system for circRNAs is currently lacking and future circRNA therapies aim to enhance specificity and efficacy through targeted systemic delivery methods based on tissue-specific expression. The delivery efficiency and targeting of these molecules can be enhanced through artificial modifications of nanoparticles and exosomes. This strategy necessitates further validation in future research. Moreover, the design of engineered exogenous circRNAs incorporating ORF for a specific gene, along with an IRES element to enable cap-independent translation, could represent a promising strategy for future applications in gene therapy. However, investigating the translation of circRNAs into proteins presents difficulties in terms of detecting the resulting proteins and peptides due to existing technical constraints. Consequently, there is a need for more sensitive methodologies than those currently available in proteomics. Meanwhile, it is essential to investigate whether the proteins and peptides generated by circRNAs are significant in various biological and physiological functions.

Cardiac fibrosis is a complex physiological and pathological process, and its related research is also facing some difficulties. One potential challenge is accurately identifying fibroblasts, which can be complicated by various subpopulations and the high plasticity associated with these cells. With the aid of increasingly advanced technologies such as lineage tracing and single-cell sequencing, there are new insights into the heterogeneity of these cells, which will further facilitate the research on personalized treatment of cardiac fibrosis. Cardiac fibrosis exhibits significant heterogeneity, arising from a wide range of etiological factors and encompassing multiple stages within the fibrotic process, leading to diverse molecular expressions and clinical outcomes. It is advisable to pursue an accurate and precise diagnosis of cardiac fibrosis, and the search for novel biomarkers is advancing, as provided by molecular imaging and -omics.

The research on the molecular mechanisms that underpin circRNAs’ ability to function has become increasingly thorough. The comprehension of circRNAs has also expanded in scope. Most studies have investigated the involvement of circRNAs in regulating miRNAs in cardiac fibrosis. Regarding research on this molecular mechanism, emphasis should be placed on quantifying circRNAs compared with miRNAs. Typically, the expression of circRNAs is minimal, demanding careful consideration of their potential impact on the quantity of self-free miRNAs in the cell through its adsorption of specific miRNAs. Until now, investigations into other molecular mechanisms have been limited, and clinical research on circRNAs in CVDs, specifically cardiac fibrosis, is sparse. Further endeavors are required to upgrade technologies and equipment to investigate the molecular mechanisms behind circRNAs in cardiac fibrosis. With the continuous advancements in the detection and precise modulation of circRNAs, enhancements in delivery systems, innovations in circRNA engineering, and a deeper understanding of cardiac fibrosis, it is expected that research into circRNAs as potential biomarkers or therapeutic targets for addressing the advancement of cardiac fibrosis will experience progress in the foreseeable future.

## CRediT authorship contribution statement

**Qianhui You:** Writing – original draft, Formal analysis, Conceptualization. **Jiajing Yu:** Validation, Project administration. **Runfang Pan:** Investigation. **Jiaming Feng:** Investigation. **Haidong Guo:** Writing – review & editing. **Baonian Liu:** Writing – review & editing, Conceptualization.

## Ethics approval and consent to participate

Not applicable.

## Consent for publication

Not applicable.

## Availability of data and materials

All data generated or analyzed during this study are included in this article.

## Funding

This work was supported by grants from the 10.13039/501100001809National Natural Science Foundation of China (82204831, 82174120), the Natural Science Foundation of Shanghai (No. 21ZR1463100), the 10.13039/501100012247Program of Shanghai Academic Research Leader (22XD1423400), the Shanghai Sailing Program (No. 22YF1448800), and the 10.13039/501100002858China Postdoctoral Science Foundation (No. 2021M692153).

## Declaration of competing interest

The authors declare the following financial interests/personal relationships which may be considered as potential competing interests: None
